# glorund functions in the *Drosophila* intestine to regulate triglyceride storage and the expression of lipid transport protein genes

**DOI:** 10.17912/micropub.biology.002017

**Published:** 2026-02-25

**Authors:** Roman Voskoboynikov, Justin R. DiAngelo

**Affiliations:** 1 Division of Science, Penn State Berks, Reading, PA

## Abstract

The intestine acts as the primary site for absorption of dietary lipids. These lipids are packaged and transported via lipoprotein complexes, whose altered levels correlate with metabolic disease. &nbsp;The *Drosophila* splicing factor glorund (glo) has been shown to affect the expression of apoB-family lipoproteins, including microsomal triacylglycerol transfer protein, lipid transfer particle, and lipophorin, in the fly adipose tissue. Here, we demonstrate that decreasing *glo* in intestines leads to increased whole animal triglyceride storage, but decreased expression of lipid transport protein genes. Together, these data suggest that glo functions in the intestine to regulate lipid transport and organismal fat storage.

**Figure 1. Intestinal knockdown of glo increases overall triglycerides and decreases lipid transport gene expression:**
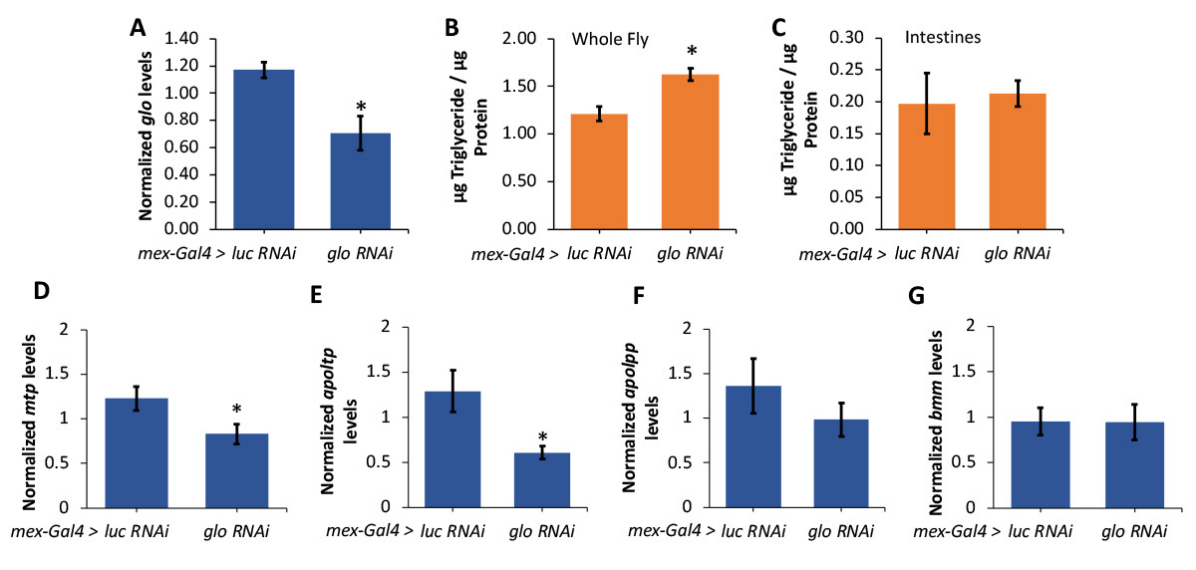
**A.** Levels of *glo* expression were measured in RNA isolated from intestines dissected from one-week old female *mexGal4>luc RNAi* and *mexGal4>glo RNAi* flies and normalized to *rp49* levels (n=6). Triglyceride normalized to total protein content was measured in **B.** whole flies (n=22) and **C.** intestines (n=17) dissected from one-week old female *mexGal4>luc RNAi* and *mexGal4>glo RNAi* flies. Levels of **D.** *mtp* , **E.** *apoltp* , **F.** *apolpp* , and **G.** *bmm* were measured using qPCR on RNA isolated from intestines dissected from one-week old female *mexGal4>luc RNAi* and *mexGal4>glo RNAi* flies and normalized to *rp49* levels (n=6). Bars indicate average, +/- standard error. *P<0.05 by a t-test comparing *mexGal4>luc RNAi* and *mexGal4>glo RNAi* conditions.

## Description

After a meal, dietary triglycerides (TAGs) are incorporated into lipoprotein molecules called chylomicrons in the intestine and then carried to muscle and adipose tissue, where they are hydrolyzed releasing free fatty acids, which are subsequently esterified into TAGs for storage in adipose tissue, or metabolized to produce energy in muscle (Mahmood, 2014). The microsomal TAG transport protein (MTP), which is expressed in the liver and intestinal mucosa, catalyzes the transfer of dietary TAGs and cholesteryl esters into chylomicrons and very low-density lipoproteins (Mahmood, 2014). *MTP* deficiency in humans can lead to a rare disease called Abetalipoproteinemia and deleting *mtp* in the mouse intestine results in decreased chylomicron assembly and secretion (Xie et al, 2006). However, how the *mtp* gene and its function are regulated is not fully understood.

Recent work from our lab and others in both humans and the genetic model system *Drosophila* has identified mRNA splicing factors as regulating lipid metabolism (Gingras et al, 2014, Pihlajamäki et al, 2011). Our lab has also shown that the *Drosophila* splicing factor *glorund* ( *glo* ) promotes lipid transport by stimulating the expression of *mtp* and other apoB-containing lipoprotein genes, *apolipophorin* ( *apolpp* ) and *apolipoprotein* *lipid transfer particle* ( *apoltp* ) (Kolasa et al, 2021). Lipids and sterols are added to the lipophorins, the major lipid carrier in flies, by LTP proteins and then secretion of the lipidated lipophorins occurs with the help of MTP molecules (Palm et al, 2012). Consistent with a role of *glo* in regulating lipid transport, decreased expression of *glo* in *Drosophila* adipose tissue results in increased TAGs stored in fat cells and less TAGs stored in non-fat body tissues (Kolasa et al, 2021). However, the function of *glo* in the *Drosophila* intestine to regulate lipid transport and metabolism is not yet known.

To study the function of *glorund* in the intestine to regulate lipid metabolism, *glo* expression was decreased in *Drosophila* intestines by inducing RNAi using the *Gal4-UAS* system ( Fig. 1A ). To determine whether *glo* function in the intestine is necessary for triglyceride storage in the entire animal, we measured triglycerides in the whole fly after intestine-specific *glo* knockdown ( Fig. 1B ). Total fly body triglycerides were increased in *glo-RNAi* flies suggesting glorund acts in the fly intestine to regulate overall lipid storage. To investigate whether decreasing *glorund* altered intestinal lipid storage autonomously, intestines were dissected from *glo-RNAi* flies and triglycerides were analyzed ( Fig. 1C ). Interestingly, triglyceride storage was not altered in the *glo-RNAi* intestines. However, triglycerides still accumulate throughout the entire fly after *glo* knockdown, which suggests that glorund may act in the *Drosophila* intestine to regulate lipid transport.

To determine if the decrease of *glo* is affecting the lipid transport machinery, RNA was isolated from *glo-RNAi* intestines and qPCR was performed to detect the levels of several key lipoprotein genes involved in lipid transport ( *mtp* , *apoltp* , and *apolpp* ), and the triglyceride lipase *bmm* . Interestingly, *mtp* and *apoltp* were both significantly lower in intestines with decreased *glo* levels ( Fig. 1D- 1E), while *apolpp* and *bmm* expression was not changed ( Fig. 1F- 1G). Together, these data suggest that glorund may function in the fly intestine to regulate the storage and transport of lipids.

In this study, we have shown that the splicing factor, glorund, plays a role in the *Drosophila* intestine to regulate lipid storage, perhaps through controlling lipid transport as the expression of the apoB-containing genes *mtp* and *apoltp* are decreased in *glo-RNAi* flies.&nbsp; Previous studies decreasing *mtp* levels specifically in the intestine in mice has resulted in decreased lipid absorption and chylomicron secretion leading to triglyceride buildup in the intestine (Xie et al, 2006, Iqbal et al, 2010, Iqbal et al, 2014).&nbsp; In addition, intestine-specific *mtp* knockout mice had increased hepatic lipogenesis, perhaps compensating for the decreased lipid absorption and transport from the intestine (Xie et al, 2006).

While we did not observe triglyceride accumulation in *glo-RNAi* intestines, it is possible that lipid transport could be decreased in these animals and measuring apoB-containing protein secretion from the intestine as well as lipid profiles in the hemolymph of *glo-RNAi* flies may help to further characterize any lipid transport defects that may exist in these flies.&nbsp; Moreover, the increased triglycerides in *glo-RNAi* animals could be due to increased lipogenesis in fat body tissue like what was seen in livers of intestine-specific *mtp* knockout mice (Xie et al, 2006); measuring lipogenesis in fat tissue in *glo-RNAi* flies would help test this hypothesis. &nbsp;In addition, it is also possible that additional genes to those shown here are regulated by glorund to control lipid storage; further experimentation measuring differential gene expression in flies with decreased *glo* expression would help characterize how the glorund protein regulates lipid storage and metabolism in the fly intestine.

## Methods

Fly husbandry:&nbsp; Flies were cultured on standard yeast-sugar-cornmeal medium (9 g *Drosophila* agar (Genesee Scientific), 100 mL Karo Lite Corn Syrup, 65 g cornmeal, 40 g sucrose, and 25 g whole yeast in 1.25 L water) and grown in an incubator at 25°C with a 12h:12h light:dark cycle. *UAS-lucRNAi* and *UAS-gloRNAi* males were crossed to *mex-Gal4* virgin females and approximately 1 week old, mated female progeny were used in the experiments described here.

Triglyceride and protein assays:&nbsp; Whole one-week old female fly pairs, or groups of 10 intestines dissected from one-week old females were suspended in lysis buffer (140 mM NaCl, 50 mM Tris-HCl, pH 7.4, 0.1% Triton-X, and 1X protease inhibitor (Roche)), and then homogenized via sonication. Protein concentrations were measured using the Pierce BCA Assay kit (Thermo Fisher Scientific) and triglyceride concentrations were measured using the Infinity Triglyceride Reagent (Thermo Fisher Scientific) according to manufacturer’s instructions. Triglyceride concentrations were normalized to protein levels for each sample.

RNA isolation: Groups of 25-30 intestines dissected from one-week old female flies were suspended in TRIzol Reagent (Thermo Fisher Scientific) and homogenized briefly followed by a 5 min incubation at room temperature. Samples were chloroform extracted and nucleic acids were precipitated by adding isopropanol. Pellets were washed with 70% ethanol, air dried for 5 minutes, and then resuspended in nuclease-free water.

DNase treatment and cDNA synthesis: 5 µg of each RNA sample was DNase treated with the DNA-Free Kit (Ambion), according to manufacturer’s instructions. 0.25 µg of DNased RNA samples were reverse transcribed using qScript Ultra cDNA Supermix (QuantaBio), according to manufacturer’s instructions.

qPCR: qPCR reactions were made from 1µl of cDNA, 2x Perfecta SYBR Green (QuantaBio) and 200nM of the forward and reverse primer for each gene segment. The qPCR cycling conditions were as follows: 3 min at 95 °C; 40 cycles of: 30 s at 95 °C, 1 min at 60 °C, and 30 s at 72 °C, with a melt curve. The following genes were amplified as described in Kolasa et al (2021): *glo,* *rp49* , *apoltp* , *mtp* , *apolpp* , and *bmm* . Resulting values for each gene were normalized to *rp49* .&nbsp; The primer sequences are shown below.

**Table:** 

**Primer**	**Forward Sequence (5’->3’)**	**Reverse Sequence (5’-> 3’)**
*glo* ( *CG6946* )	GCTGGGCTTCAACAATCTGC	AATTTCCACCGTTGTTGCCG
*rp49* ( *CG7939* )	GACGCTTCAAGGGACAGTATCTG	AAACGCGGTTCTGCATGAG
*apoltp* ( *CG15828* )	GTTCGAGGTGAGTGGTTGGT	AGCTGCGTCTCATTGGAGAT
*apolpp* ( *CG11064* )	ATCGGCTCAACACAAAAACC	AGGCAAAAGCGATCTCAAAA
*mtp* ( *CG9342* )	GTGGGAAGCTTCGTGAAGAG	AAAACGCGATACCATTCGAG
*bmm* ( *CG5295* )	ACGTGATCATCTCGGAGTTTG	ATGGTGTTCTCGTCCAGAATG

## Reagents

**Table:** 

**Fly Line**	**Genotype**	**Source**
UAS-luc RNAi	*UAS-lucRNAi y[1] v[1]; P{y[+t7.7] v[+t1.8]=UAS-LUC.VALIUM10}attP2*	BL#35788
UAS-glo RNAi	UAS-gloRNAi y[1] sc[*] v[1] sev[21]; P {y[+t7.7] v[+t1.8]=TRiP.HMS00079}attP2	BL#33668
Mex-Gal4	*w[1118]; P{w[+mC]=mex1-GAL4.2.1}10-8*	BL#91368
